# Target Specificity of the E3 Ligase LUBAC for Ubiquitin and NEMO Relies on Different Minimal Requirements* [Fn FN2]

**DOI:** 10.1074/jbc.M113.495846

**Published:** 2013-09-12

**Authors:** Judith J. Smit, Willem J. van Dijk, Dris El Atmioui, Remco Merkx, Huib Ovaa, Titia K. Sixma

**Affiliations:** From the ‡ Division of Biochemistry and; § Division of Cell Biology II, The Netherlands Cancer Institute, 1066 CX Amsterdam, The Netherlands

**Keywords:** E3 Ubiquitin Ligase, Enzyme Mechanisms, Molecular Biology, NF-κB (NF-KB), Ubiquitination, LUBAC, Linear Ubiquitin Chain, RNF31

## Abstract

The ubiquitination of NEMO with linear ubiquitin chains by the E3-ligase LUBAC is important for the activation of the canonical NF-κB pathway. NEMO ubiquitination requires a dual target specificity of LUBAC, priming on a lysine on NEMO and chain elongation on the N terminus of the priming ubiquitin. Here we explore the minimal requirements for these specificities. Effective linear chain formation requires a precise positioning of the ubiquitin N-terminal amine in a negatively charged environment on the top of ubiquitin. Whereas the RBR-LDD region on HOIP is sufficient for targeting the ubiquitin N terminus, the priming lysine modification on NEMO requires catalysis by the RBR domain of HOIL-1L as well as the catalytic machinery of the RBR-LDD domains of HOIP. Consequently, target specificity toward NEMO is determined by multiple LUBAC components, whereas linear ubiquitin chain elongation is realized by a specific interplay between HOIP and ubiquitin.

## Teaser

**Background:** Linear ubiquitination of NEMO by LUBAC is important for NF-κB activation.

**Results:** HOIP and the “top” of ubiquitin are essential for linear ubiquitination, whereas NEMO ubiquitination additionally requires HOIL-1L.

**Conclusion:** NEMO priming and ubiquitin chain elongation rely on different LUBAC contributions.

**Significance:** Novel insights in the requirements for linear ubiquitin chain formation and target selection.

## Introduction

The nuclear factor of κ-B (NF-κB) is a transcription factor that plays a central role in inflammatory and immune responses (1, 2). Its activation is regulated by a variety of post-translational modifications, including phosphorylation and various types of ubiquitination. The formation of Lys-63-linked and linear ubiquitin chains, which are linked via the ubiquitin N terminus, are crucial for the activation of the canonical NF-κB pathway (3–7). Upon TNF-receptor activation, RIP1 is ubiquitinated at the receptor with Lys-63 and linear ubiquitin chains, which leads to the recruitment of the IKK-complex that consists of NF-κB essential modulator (NEMO^2^, also known as IKKγ), IKKα, and IKKβ. Subsequently, NEMO is ubiquitinated with linear ubiquitin chains that increase the efficiency by which IKKβ is phosphorylated and activated (8, 9). The activation of IKKβ leads to the phosphorylation and subsequent degradation of the inhibitor of NF-κB, IκBα, which enables the NF-κB proteins p50 and p65 to translocate to the nucleus and induce anti-apoptosis and inflammatory responses (4, 8, 10, 11). Consequently, linear ubiquitin chain formation is a key early event in the activation of the pathway.

Ubiquitin chains consist of multiple ubiquitins that are typically linked via the donor ubiquitin C terminus to any of the seven lysine residues on the target ubiquitin, but in linear ubiquitin chains the N-terminal amine of the target ubiquitin is used (11, 12). Depending on which target site is used in a ubiquitin chain, the ubiquitination of proteins leads to different cellular outcomes, such as proteasomal degradation and intracellular translocation. Linear ubiquitin chains are essential for the activation of the NF-κB pathway by acting as interaction sites for NEMO and HOIL-1L (13–15). However, they also recruit the negative regulator of NF-κB, A20, illustrating the dual role of this posttranslational modification (16, 17).

The formation of ubiquitin chains is mediated by a cascade of E1-E2-E3 enzymes (18–20). A donor ubiquitin is activated in an ATP-dependent manner by an E1, after which the thioester bond that is formed between the ubiquitin C terminus and a cysteine on the E1 is transferred onto the active site cysteine of an E2. The final conjugation of the ubiquitin C terminus onto its target is mediated by E3 ligases. Two major classes of E3-ligases are the RING- and HECT-type E3s. RING E3-ligases indirectly mediate the transfer of the ubiquitin by interacting with the E2 and the target, whereas HECT E3 ligases form a thioester intermediate with the ubiquitin during the transfer onto a target. The novel class of RING-between-RING (RBR) E3 ligases contains three zinc-finger domains (RING1, IBR, RING2) in a conserved unit (21–26), which mediate ubiquitin chain formation by a combined RING/HECT type mechanism (27–29). The first RING domain of the RBR interacts with the E2 to facilitate the formation of a HECT-type intermediate between the ubiquitin and an active-site cysteine in the RING2 domain before it is transferred onto its target (27, 28).

The ubiquitination of NEMO with linear ubiquitin chains is performed by the E3-ligase linear ubiquitin chain assembly complex (LUBAC) (4). LUBAC consists of the proteins HOIP, HOIL-1L, and Sharpin (4, 9, 11, 30, 31), of which HOIP and HOIL-1L belong to the RBR class of E3-ligases (28, 29). Even though both HOIP and HOIL-1L have an RBR domain, HOIP is the catalytic subunit of the complex (28, 29). The linear ubiquitin chain-forming activity and specificity of LUBAC is completely embedded within HOIP, which is the only E3 ligase that is known to build linear ubiquitin chains. HOIP catalyzes the specific linear ubiquitin chain formation by transferring the ubiquitin from its active site Cys-885 on RING2 to the N terminus of the target ubiquitin that is positioned by its unique C-terminal linear ubiquitin chain determining domain (LDD) (28, 29).

**FIGURE 1.**
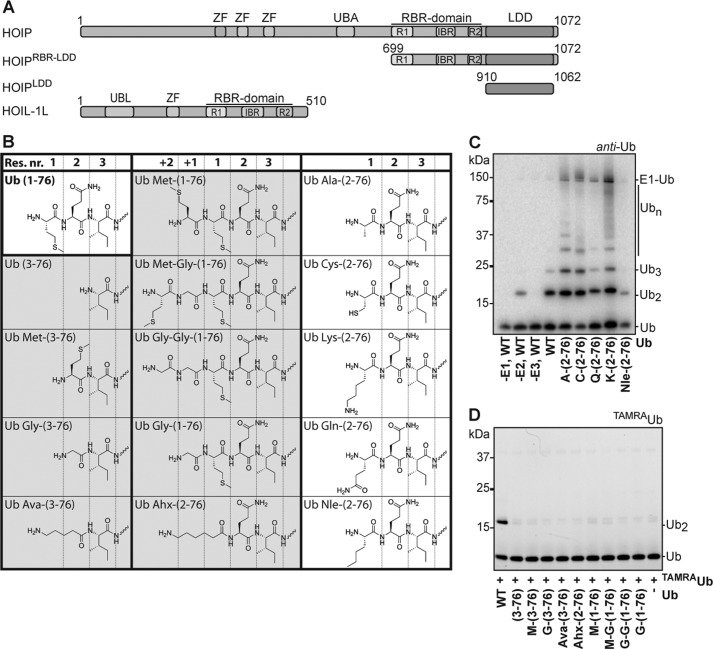
**HOIP^RBR-LDD^ mediated ubiquitin chain formation with N-terminal modified synthetic ubiquitins.** *A*, E3 ligase constructs used in this study. HOIP ubiquitin-like domain (*UBL*), Npl4 zinc finger (*ZF*), ubiquitin-associated domain (*UBA*), LDD, and a RBR consisting of two RING domains (*R1* and *R2*) and an in-between RING domain (*IBR*). The domain borders of the ubiquitin-like domain, ZF, UBA, and RBR domains are drawn to scale according to Uniprot definitions. *B*, schematic representation of the N-terminal residues of the synthetic N-terminal-modified ubiquitins. Construct names represent the wild type amino acids between *parentheses* and additional amino acids by their three-letter code. Norleucine (*Nle*) was used as a steric equivalent of Met-1, and 5-aminovaleric acid (*Ava*), 6-aminohexanoic acid (*Ahx*) were designed to position an amino group at a similar position compared with the wild type N terminus. Ubiquitin mutants that were not used by HOIP have a *gray background. C*, HOIP^RBR-LDD^ (E3) functions together with Ube2L3 (E2) to mediate free ubiquitin chain formation with ubiquitin Met-1 point mutants after 30 min. *D*, acceptor assay with ^TAMRA^ubiquitin loaded on the E2 Ube2L3. HOIP^RBR-LDD^ does not transfer ^TAMRA^ubiquitin from the E2 onto the N-terminally shortened or elongated ubiquitins.

The linear ubiquitin chain-forming activity of HOIP is autoinhibited by its N terminus (28, 29). To release the inhibited state, full-length HOIP needs to form a complex via its UBA domain with the UBL domain of either HOIL-1L or Sharpin (32). The different HOIP-containing complexes that consist of either HOIP·HOIL-1L or HOIP·Sharpin or HOIP·HOIL-1L/Sharpin can all activate the NF-κB pathway (4, 9, 30, 31). However, the isolated HOIP RBR-LDD domain, which lacks the HOIP N terminus, is sufficient for the formation of free linear ubiquitin chains *in vitro* in the absence of the other LUBAC components (Fig. 1*A*) (28, 29).

The formation of linear ubiquitin chains on NEMO by LUBAC requires the “priming” of the first ubiquitin on a NEMO lysine and ubiquitin chain formation on the ubiquitin N terminus, two reactions with different chemistries. Currently, it is unknown how this dual target specificity is regulated. We analyzed free linear ubiquitin chain formation and NEMO modification *in vitro* to gain insight into the minimal requirements of LUBAC and ubiquitin that are needed for the reactions.

## EXPERIMENTAL PROCEDURES

### 

#### 

##### Construction of Plasmids

*Escherichia coli* expression constructs of HOIP, HOIP^RBR-LDD^, and HOIL-1L have been described previously (28). Full-length HOIP C885A and C916A were subcloned from the previously described pcDNA3.1-Myc-HOIP into pGEX-6P-1 vectors (GE Healthcare) with an N-terminal GST tag for *E. coli* expression (28). The full-length HOIL-1L^C460A^ point-mutant was introduced in a HOIL-1L pGEX-6P-1 construct with the QuikChange Mutagenesis kit from Stratagene (La Jolla, CA). The pASK-IBA3plus Strep-NEMO^242–419^ expression construct was kindly provided by Prof. Dr. D. Krappmann (Helmholtz Zentrum München) (33). The pGex5X GST-NEMO full-length expression construct was kindly provided by Prof. Dr. K. Iwai (Osaka University) (34). Ubiquitin single point mutations were introduced in a pET3a-ubiquitin construct by using the QuikChange mutagenesis kit from Stratagene.

##### Protein Expression and Purification

Ubiquitin, hUba1, Ube2L3, Ube2N/Ube2V2, HOIP^RBR-LDD^, and HOIL-1L were expressed and purified as described previously (28, 35–38). Purification of full-length HOIP was as described previously, modified by using a Bead Beater (Mixer Mill MM400, Retsch) for cell lysis (28). Strep-NEMO^242–419^ was expressed in *E. coli* Bl21 (DE3) pLysS cells by induction with 0.8 mm IPTG overnight at 18 °C. Cells were resuspended in 20 mm Tris/HCl, pH 8.0, 100 mm NaCl, 5 mm β-mercaptoethanol (βME) and Complete EDTA-free protease inhibitor mixture (Roche Applied Science). Cells were lysed by a high pressure EmulsiFlex-C5 device (Avestin, Mannheim, Germany). Initial purification was achieved by binding the protein to StrepTactin high performance resin (GE Healthcare) and elution in buffer containing 2.5 mm desthiobiotin. The protein was further purified over a Resource Q column followed by gel filtration (Superdex 75) in 20 mm Hepes/HCl, pH 8, 150 mm NaCl, and 5 mm βME.

GST-NEMO was expressed in *E. coli* Rosetta (DE3) cells by induction with 0.5 mm isopropyl-1-thio-β-d-galactopyranoside overnight at 18 °C. Cells were resuspended in 50 mm Hepes/HCl, pH 8.0, 150 mm NaCl, 10 mm MgCl_2_, 5 mm βME supplemented with DNase1 and Complete EDTA-free protease inhibitor mixture (Roche Applied Science). Cells were lysed by a high pressure EmulsiFlex-C5 device (Avestin). The cleared lysate was incubated with glutathione beads (GE Healthcare), and the GST-tagged protein was eluted in buffer supplemented with 50 mm GSH. The protein was further purified over a Heparin column followed by gel filtration (Superose 6) in 50 mm Hepes/HCl, pH 8, 150 mm NaCl, and 5 mm βME.

##### Ubiquitin Synthesis

Synthetic ubiquitin, synthetic ubiquitin N-terminal variants, and ^TAMRA^ubiquitin were synthesized according to El Oualid *et al.* (36) and subsequently purified over a Resource S and gel filtration (Superdex 75) according to the same protocol as for wild type ubiquitin.

##### LC-MS Analysis of Synthetic Ubiquitins

LC-MS measurements were performed on a system equipped with a Waters 2795 Separation Module (Alliance HT), Waters 2996 Photodiode Array Detector (190–750 nm), Phenomenex Kinetex C18 (2.1 × 50, 2.6 μm) column, and LCTTM Orthogonal Acceleration Time of Flight Mass Spectrometer. Samples were run using 2 mobile phases: A = 1% CH_3_CN, 0.1% formic acid in water, and B = 1% water and 0.1% formic acid in CH_3_CN; flow rate = 0.8 ml/min; run time = 6 min; column T = 40 °C. Gradient: 0–0.5 min, 5% B; 0.5–4 min, à 95% B; 4–5.5 min, 95% B. All synthetic peptides eluted as a single peak; data processing was performed using Waters MassLynx Mass Spectrometry Software 4.1 (deconvulation with Maxent1 function).

##### In Vitro Ubiquitin Chain Formation

*In vitro* ubiquitination reactions were performed under standard conditions containing 100 nm hUba1, 600 nm Ube2L3 (unless indicated otherwise), 1 μm E3, 1 μm NEMO, 20 μm ubiquitin, and 10 mm ATP in buffer containing 20 mm Hepes/HCl, pH 8, 150 mm NaCl, 10 mm MgCl_2_, 5 mm βME unless specified otherwise. The GST tag of full-length NEMO was cleaved by Factor Xa (Sigma) while the ubiquitination reaction was continued overnight at 15 °C. Samples were separated on 4–12% NuPAGE gels (Invitrogen) in MES buffer and analyzed by Western blot using mouse-anti-ubiquitin antibody (P4D1, Santa Cruz Biotechnology) and HRP conjugated anti-mouse antibody (Bio-Rad), anti-Strep antibody (StrepMAB-Classic-HRP, IBA), or goat-anti-GST antibody (GE Health) and swine-anti-goat HRP-antibody (BIOSOURCE).

##### Donor/Acceptor Assays

Donor/acceptor assays were performed in the same buffer conditions as described for the ubiquitin chain formation. N-terminal TAMRA-labeled (500 nm) or N-terminally modified ubiquitin (10 μm) was loaded onto Ube2L3 (600 nm/1 μm) in the presence of ATP (1 mm) and hUba1 (100 nm) for 15 min at 37 °C. Subsequently, HOIP^RBR-LDD^ (1 μm) and target ubiquitin (500/10 μm) were added to the reactions and incubated for 20 min. The reactions were stopped by the addition of protein loading buffer. Samples were analyzed on 4–12% NuPAGE gels (Invitrogen) in MES buffer followed by Western blotting, or in the case of ^TAMRA^ubiquitin, the TAMRA signal was visualized on a ChemiDoc XRS (Bio-Rad).

##### Fluorescence Polarization Assays

The fluorescence anisotropy of N-terminal TAMRA-labeled ubiquitin (1 nm) in binding buffer (20 mm Hepes, pH 7.5, 150 mm NaCl, 5 mm βME, and 1 g/liter chicken ovalbumin) was measured on a PHERAstar FS (BMG Lab Tech). The 1:1 serial dilutions were performed in three repeats. The binding was measured in the 30-μl samples with 540-nm excitation and 590-nm emission, with correction for both the buffer background and G-factor of the instrument. The resulting binding isotherms (anisotropy *versus* HOIP^LDD^ concentration) were fit to a 1:1 non-linear binding model (*Y* = *B*_max_ × *X*/(*K_d_* + *X*)). Samples were prepared and analyzed as described previously (28).

## RESULTS

### 

#### 

##### The Side Chain of Ubiquitin Residue Met-1 Is Not Involved in Chain Formation

The interplay between the HOIP RBR-LDD domain and ubiquitin dictates the linear ubiquitin chain formation specificity of the LUBAC complex. Previously we illustrated that the LDD region of HOIP is essential for linear ubiquitination (28); now we characterize the features of the target ubiquitin that are important for the formation of these linear ubiquitin chains. The N-terminal modification of the target ubiquitin is a highly specific reaction, as the potential ubiquitination site Lys-63 that is located very close to the N terminus is not modified by HOIP. To test which aspects of the N terminus are important for this specificity, we designed and chemically synthesized ubiquitins with varying N termini, changing either the side chain of the N-terminal methionine 1 (Met-1) or the position of the N-terminal amine (Fig. 1*B*, supplemental Fig. 1, *A* and B).

To test the importance of the ubiquitin Met-1 side chain, we changed it into various natural and non-natural amino acids. The ubiquitin chain-forming activity of HOIP^RBR-LDD^ with these mutants was compared with chain formation with chemically synthesized wild type ubiquitin in *in vitro* assays. Unlike with the wild type ubiquitin, the E1 made covalent bonds with the ubiquitin M1A, -C, -Q, and -K point mutants (Fig. 1*C*, supplemental Fig. 1*C*). Nevertheless, the ubiquitin∼E2 thioester intermediate was formed on the E2 Ube2L3 (UbcH7) (supplemental Fig. 1*C*), and HOIP^RBR-LDD^ mediated ubiquitin chain formation with all Met-1 point mutants (Fig. 1*C*). The different chain-forming efficiencies are reflected by the differences in E2 loading (supplemental Fig. 1*C*). Together these results show that the methionine side chain of the first ubiquitin residue is not essential for the HOIP-mediated N-terminal modification.

##### The Position of the N Terminus on Ubiquitin Is Critical for Its Modification

We next tested the importance of the position of the N-terminal amino group within the structure of the target ubiquitin. For this purpose, we designed N-terminally extended and shortened synthetic ubiquitins and modified some of the shortened ubiquitins with chemical groups (5-aminovaleric acid or 6-aminohexanoic acid) that potentially could allow an amino group to extend as far as the normal N terminus (Fig. 1*B*, supplemental Fig. 1, *A* and B). The extended and shortened ubiquitin variants were tested in linear ubiquitin chain formation assays with HOIP^RBR-LDD^. The N-terminally shortened ubiquitins formed some E1-Ub adducts and E1-dependent di-ubiquitin that was not formed with wild type ubiquitin (supplemental Fig. 1*D*), but they were proficient for forming Lys-63-linked chains by Ube2N/Ube2V2 (Ubc13/Mms2) and could also be loaded onto the E2 Ube2L3 (supplemental Fig. 1, *C* and *E*), showing that the alterations on the ubiquitin N terminus did not impair the initial activation of the ubiquitins. However, none of these ubiquitin variants could be used by HOIP^RBR-LDD^ for ubiquitin chain formation (supplemental Fig. 1*F*).

We validated this result in donor/acceptor assays and took the opportunity to analyze whether the defect was at the level of the donor or the target ubiquitin in the reaction. For the analysis of the donor ubiquitin, the ubiquitin variants were loaded onto the E2 Ube2L3 to be discharged onto ubiquitin^ΔG76^ by HOIP^RBR-LDD^. Ubiquitin^ΔG76^ cannot be activated by the E1 because it lacks the required ubiquitin C terminus; therefore, it can only function as a target ubiquitin in the reactions. All ubiquitin mutants were covalently linked to ubiquitin^ΔG76^ by HOIP^RBR-LDD^ (supplemental Fig. 1*G*), revealing that the changes on the N terminus in these mutants do not interfere with the transfer of the donor ubiquitin from the E2 onto the target ubiquitin. Subsequently we used a similar assay to investigate if the N-terminal ubiquitin mutants could be used as target ubiquitin for linear ubiquitination. For this purpose, N-terminally labeled ^TAMRA^ubiquitin was loaded onto the E2 Ube2L3 to be discharged onto the different ubiquitin mutants. The TAMRA label of the ^TAMRA^ubiquitin is attached to the N terminus of the ubiquitin and thereby blocks the N terminus for linear ubiquitination; therefore, ^TAMRA^ubiquitin can only function as a donor ubiquitin in the reactions. In these assays only wild type ubiquitin served as a target for the ^TAMRA^ubiquitin, and none of the N-terminally modified ubiquitins was ubiquitinated by the ^TAMRA^ubiquitin (Fig. 1*D*). These results show that the N-terminally extended and shortened ubiquitin mutants are impaired as target ubiquitin for linear ubiquitination. Even ubiquitins that were designed to potentially position an amino group in the wild type position are impaired as target ubiquitin. Thus, the precise position of the N-terminal amine of ubiquitin within the target ubiquitin is essential for linear ubiquitin chain formation by HOIP.

##### E16 and E18 on the Target Ubiquitin Are Essential for Linear Ubiquitin Chain Formation

**FIGURE 2.**
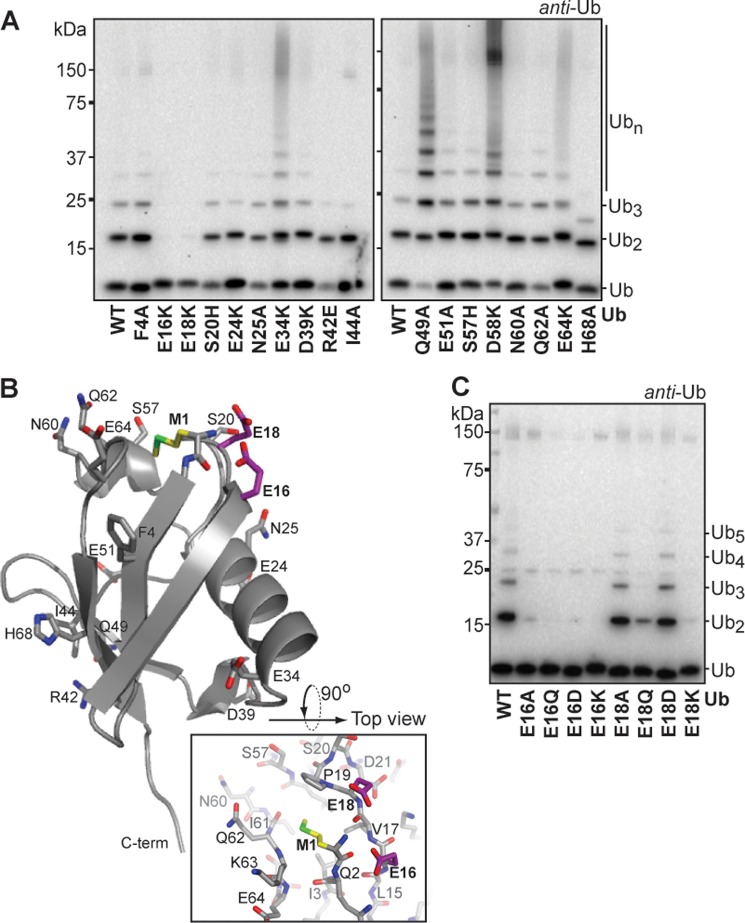
**Ubiquitin residues E16 and E18 are critical for ubiquitin chain formation.** *A*, ubiquitin chain formation by HOIP^RBR-LDD^ with different ubiquitin point-mutants in 1 h reactions. *B*, crystal structure of ubiquitin (PDB code 3PRM, chain d), illustrating the position of the tested ubiquitin surface point mutations in sticks (Met-1 (*M1*) in *yellow*, E16 and E18 in *purple*). The top view of the ubiquitin structure (PDB code 3PRM, chain d) illustrates the local environment of Met-1. *C*, HOIP^RBR-LDD^ mediated chain formation with E16 and E18 ubiquitin point mutants. Reactions were stopped after 1 h.

The restricted positioning of the N terminus within the target ubiquitin illustrates that the modification of the ubiquitin N terminus is highly specific. Because the linear ubiquitin chain formation is already mediated by the interplay between the LDD domain of HOIP and the target ubiquitin (28), we mutated the outer surface of ubiquitin to identify sites on the target ubiquitin that are important for the chain formation reaction. Previously we already showed that the ubiquitin hydrophobic patch (Leu-8, Ile-44, Val-70) is neither essential for linear ubiquitin chain formation nor for the interaction between the HOIP LDD-domain and the target ubiquitin (28). Furthermore, none of the ubiquitin lysine residues is important for LUBAC-mediated linear ubiquitin chain formation (11). Here, we test 16 additional single point mutants of the ubiquitin surface in *in vitro* ubiquitin chain formation assays with HOIP^RBR-LDD^ (Fig. 2*A*).

Most of the ubiquitin surface mutants did not impair the linear ubiquitin chain formation by HOIP^RBR-LDD^, but the ubiquitin chain formation was severely impaired with ubiquitin E16K and E18K (Fig. 2*A*). However, these mutants could be used in Lys-63-linked ubiquitin chain formation by Ube2N/Ube2V2 (supplemental Fig. 2*A*), indicating that the overall fold of the ubiquitins is fine and they can be activated and used by E1-E2 enzymes.

Ubiquitin E16 and E18 are positioned next to the ubiquitin N terminus on the top of ubiquitin, where they can form a salt-bridge with the N-terminal amine, as observed in several ubiquitin structures (Fig. 2*B*). To test the importance of ubiquitin E16 and E18 in the linear chain formation reaction, we introduced additional, less dramatic mutations at these sites, maintaining the charge (E16/18D) or replacing the glutamic acids with alanine or glutamine. All E16 and E18 point mutants could be loaded on the E2 (supplemental Fig. 2*B*), showing that the initial activation by E1-E2 was not affected. However, the HOIP^RBR-LDD^-mediated ubiquitin chain formation of ubiquitin E16A, -Q, -D, and -K and E18Q and -K was impaired (Fig. 2*C*). Interestingly, the E18A and E18D ubiquitin mutants were used by HOIP^RBR-LDD^ similarly well as wild type ubiquitin, indicating that E16 is more critical to the reaction than E18.

**FIGURE 3.**
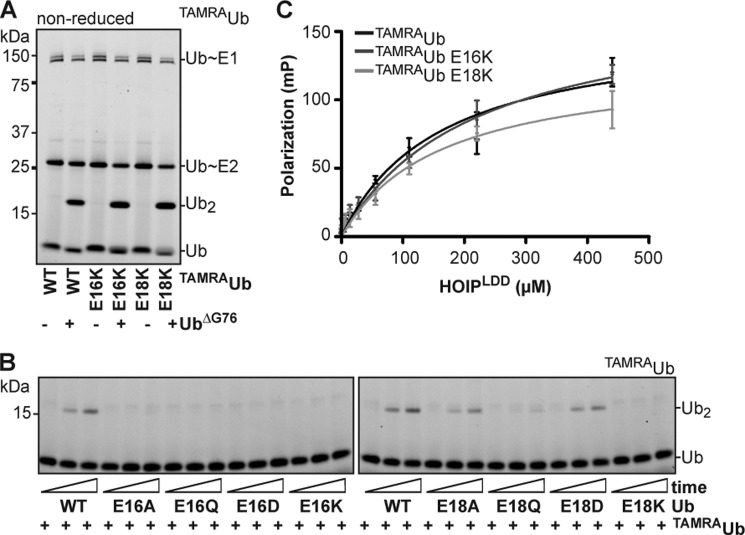
**Ubiquitin E16 and E18 are essential for the target ubiquitin.** *A*, ^TAMRA^ubiquitin wild type; E16K and E18K can be used as donor ubiquitin. All ^TAMRA^ubiquitins are discharged onto the target ubiquitin^ΔG76^. *B*, the donor ^TAMRA^ubiquitin was discharged onto different ubiquitins in an acceptor assay with HOIP^RBR-LDD^. Some of the ubiquitin E16 and E18 mutants could be used as target ubiquitin. *C*, fluorescent polarization (*FP*) assay of ^TAMRA^ubiquitin and ^TAMRA^ubiquitin mutants binding to HOIP^LDD^ showing the absolute increase in FP as a function of HOIP concentration. ^TAMRA^ubiquitin *K_D_* = 0.16 ± 0.04 mm; ^TAMRA^ubiquitin E16K *K_D_* = 0.22 ± 0.06 mm; ^TAMRA^ubiquitin E18K *K_D_* = 0.16 ± 0.05 mm.

Next we tested whether these residues affected the donor or the target ubiquitin in HOIP^RBR-LDD^-mediated ubiquitin chain formation. We used chemically synthesized N-terminal TAMRA-labeled ubiquitin mutants to investigate if ubiquitin E16 and E18 are essential for the donor ubiquitin. The ^TAMRA^ubiquitin E16K and E18K were loaded onto the E2 Ube2L3 to be discharged and covalently linked to a target ubiquitin. HOIP^RBR-LDD^ linked both ^TAMRA^ubiquitin mutants to ubiquitin^ΔG76^, showing that the donor ubiquitin is not dependent on ubiquitin E16 or E18 (Fig. 3*A*). However, the ubiquitin E16 and E18 mutants were impaired as target ubiquitin in assays with ^TAMRA^ubiquitin as the donor ubiquitin (Fig. 3*B*). The effects of the ubiquitin mutants on the target ubiquitin were the same as in the free ubiquitin chain formation assay (Fig. 2*C*), where ubiquitin E16A, -Q, -D, and -K and E18Q and -K were more impaired than the ubiquitin E18A and -D mutants. Therefore, ubiquitin E16 and to a lesser extent E18 are important on the target ubiquitin for its N-terminal modification.

The target ubiquitin interacts with the HOIP LDD domain for its ubiquitination (28). Therefore, we wondered whether E16 and E18 could be involved in the positioning of the target ubiquitin by interacting with the LDD domain. We tested if the ubiquitin E16K and E18K mutants could still bind the HOIP LDD domain in a fluorescence polarization assay with the TAMRA-labeled ubiquitin mutants. Both ubiquitin mutants bound with a similar affinity as wild type ubiquitin to HOIP^LDD^ (Fig. 3*C*), showing that the interaction between the target ubiquitin and HOIP is not dependent on ubiquitin E16 and E18.

We show that ubiquitin E16 and E18 are critical residues on the target ubiquitin in linear ubiquitin chain formation. Nevertheless, the residues are not involved in the interaction between the target ubiquitin and the HOIP LDD domain. However, ubiquitin E16 and E18 are positioned close to the ubiquitin N-terminal amino group that is targeted in chain formation. Therefore, these residues are likely to be directly involved in the catalysis of the isopeptide bond formation on the ubiquitin N terminus.

##### Targeting of NEMO Requires Multiple LUBAC Components

**FIGURE 4.**
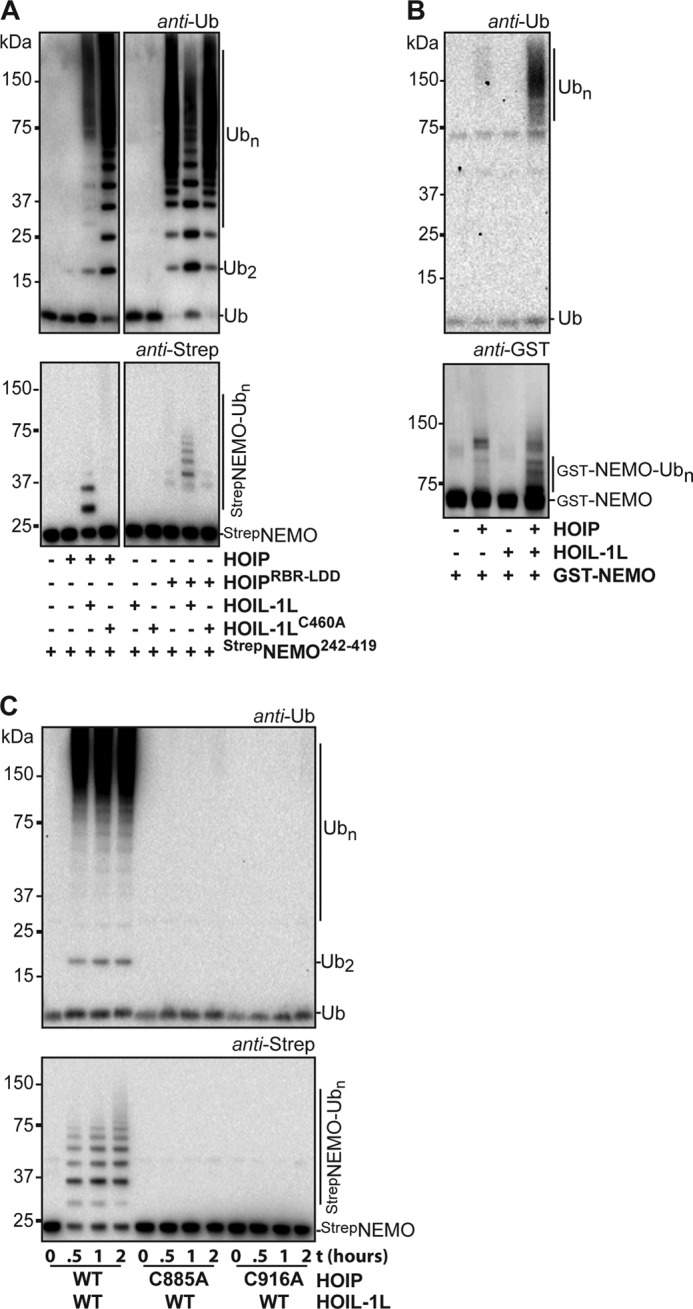
**NEMO ubiquitination requires the presence of HOIP and HOIL-1L.** *A*, linear chains are made by HOIP (*top panel*), whereas Strep-NEMO^242–419^ ubiquitination requires the addition of wild type HOIL-1L to the reaction (*bottom panel*). 1-h reactions were performed with 4 μm Strep-NEMO^242–419^ in 20 mm Hepes, pH 8, 50 mm NaCl, 1 mm MgCl_2_, 5 mm βMe. The figure shows two parts of a single Western blot. *B*, full-length HOIP and HOIL-1L ubiquitinate full-length GST-NEMO in a 1-h reaction. HOIP is slightly active in the absence of HOIL-1L. *C*, HOIP RING2 and LDD are essential for NEMO^242–419^ ubiquitination. The blots show the ubiquitin chain formation and Strep-NEMO^242–419^ modification with HOIL-1L and different HOIP mutants after 0, 0.5, 1, and 2 h.

HOIP^RBR-LDD^ mediates the formation of linear ubiquitin chains in the absence of other LUBAC components. Therefore, we wondered if HOIP^RBR-LDD^ is sufficient for the *in vitro* ubiquitination of the target NEMO. The *in vitro* ubiquitination assays were set up with a short Strep-NEMO^242–419^ construct, which includes the known minimal domain of NEMO (amino acids 241–344) that is required for its ubiquitination on Lys-285 and Lys-309 (4). We compared the activity of HOIP^RBR-LDD^ to the activity of full-length HOIP·HOIL-1L, which forms a known minimal complex for NEMO ubiquitination in cells (4). The full-length HOIP·HOIL-1L complex and HOIP^RBR-LDD^ formed free ubiquitin chains in solution, but only the combination of HOIP and HOIL-1L ubiquitinated the substrate Strep-NEMO^242–419^ (Fig. 4*A*). We validated this target ubiquitination *in vitro* on full-length NEMO (Fig. 4*B*, supplemental Fig. 3). Unlike for Strep-NEMO^242–419^, HOIP had a minor ubiquitination activity for GST-NEMO in the absence of HOIL-1L, but both HOIP and HOIL-1L were required for the efficient modification of the full-length protein (Fig. 4*B*). This modification was directed toward lysines on NEMO and not toward the N-terminal affinity tag (supplemental Fig. 3). Interestingly, the free ubiquitin chain-forming activity of the constitutively active HOIP^RBR-LDD^ was inhibited by HOIL-1L. Concurrently the combination of HOIP^RBR-LDD^ and HOIL-1L did target Strep-NEMO^242–419^ even though the HOIP UBA domain that is needed for the HOIP·HOIL-1L interaction and the N-terminal HOIP zinc fingers that interact with NEMO are not present in HOIP^RBR-LDD^ (Fig. 4*A*) (4, 32). Therefore, HOIL-1L does not only activate the RBR in full-length HOIP, but it also regulates its free ubiquitin chain-forming activity and directs the donor ubiquitin to lysines on target proteins.

Even though HOIL-1L plays a role in the targeting of NEMO with ubiquitin, the catalytic activity of the complete HOIP RBR-LDD domain is also essential for the priming of NEMO with the first ubiquitin. The active-site cysteine HOIP RING2 mutant (C885A) and a LDD mutant (C916A) did not transfer ubiquitin onto NEMO (Fig. 4*C*). These mutations, however, do not disrupt the HOIP·HOIL-1L interaction, and the thioester intermediate on HOIP RING2 can still be formed with the HOIP C916A mutant (28), demonstrating that the active site, including the LDD region, of HOIP is required for the NEMO priming event. These results show that the priming ubiquitination of NEMO is reliant on the catalytic site of HOIP and requires subsequent contributions of HOIL-1L.

We next mutated the predicted active site cysteine in the HOIL-1L RING2 domain to alanine (C460A) to test the importance of the HOIL-1L RBR domain for the targeting of NEMO with ubiquitin. HOIL-1L^C460A^ did not mediate the ubiquitination of Strep-NEMO^242–419^, indicating that the HOIL-1L RING2 domain is important for this function (Fig. 4*A*). Interestingly, unlike HOIL-1L, HOIL-1L^C460A^ did not inhibit the free ubiquitin chain formation of HOIP^RBR-LDD^. Also, HOIL-1L^C460A^ activated the free ubiquitin chain formation activity of full-length HOIP more than wild type HOIL-1L. These results indicate that HOIL-1L counterbalances the free ubiquitin chain formation catalysis of HOIP. Possibly, the HOIL-1L cysteine competes with the target ubiquitin for the donor ubiquitin that is loaded on the active site cysteine of HOIP. However, we were unable to trap a HOIL-1L∼ubiquitin intermediate or to load HOIL-1L with ubiquitin-propargylglycine 76 (data not shown). Alternatively, the RBR of HOIL-1L might physically block the transfer of the donor ubiquitin from HOIP RING2 onto its target ubiquitin. In sum, our results show that HOIL-1L is involved in the selection of NEMO as a target for linear ubiquitination. Furthermore, besides activating the RBR of full-length HOIP, HOIL-1L limits the transfer of the donor ubiquitin from HOIP RING2 onto target ubiquitins by either providing HOIL-1L C460 as an alternative ubiquitin acceptor site or by blocking its transfer onto target ubiquitins.

##### The Priming Ubiquitination of NEMO Requires the Ubiquitin N Terminus

**FIGURE 5.**
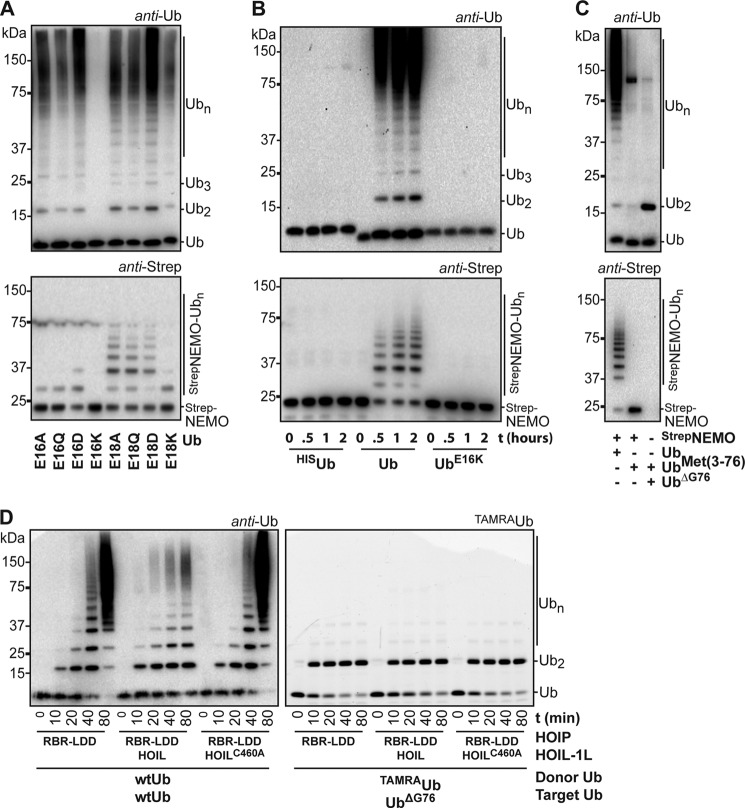
**NEMO ubiquitination requires the top of the priming ubiquitin for NEMO.** *A*, full-length HOIP·HOIL-1L mediated free ubiquitin chain formation and Strep-NEMO^242–419^ ubiquitination with different ubiquitin E16 and E18 point mutants. *B*, full-length HOIP·HOIL-1L does not mediate free ubiquitin chain formation and Strep-NEMO^242–419^ ubiquitination with N-terminally HIS-tagged ubiquitin and ubiquitin E16K. *C*, ubiquitin Met-(3–76) cannot be used by full-length HOIP·HOIL-1L to prime NEMO, but it can be linked to the target ubiquitin^ΔG76^. *D*, HOIL-1L inhibits the transfer of wild type ubiquitin onto target ubiquitins. If the N terminus is not available (^TAMRA^Ub, *right panel*), this interferes with of HOIL-1L with the transfer of the donor ubiquitin is no longer observed.

The NEMO ubiquitination assays showed that even at short time points, multiple ubiquitins could be transferred onto the target. Mechanistically this could indicate either a slow priming event of the first ubiquitin followed by highly processive chain formation or en-block transfer of ubiquitin chains onto NEMO. In slower reactions, however, with the relatively inefficiently used ubiquitin E16 and E18 mutants, it is clear that single ubiquitins are transferred onto NEMO, indicating that en bloc transfer is not required (Fig. 5*A*). These results show that the *in vitro* ubiquitination of NEMO is limited by the priming ubiquitination event on NEMO, explaining why mono-ubiquitinated NEMO is a minority of the NEMO population in most of the assays.

Surprisingly, ubiquitin E16K was not transferred onto NEMO, while it could be used as a donor ubiquitin in linear ubiquitin chain formation (Fig. 3*A* and Fig. 5, *A* and *B*), suggesting that the modification of NEMO requires different features from the donor ubiquitin than linear ubiquitin chain formation. The importance of the top of ubiquitin for the priming of NEMO was confirmed by the fact that the HOIP·HOIL-1L complex did also not transfer N-terminally HIS-tagged ubiquitin or ubiquitin Met-(3–76) (as described in the legend to Fig. 1) onto NEMO (Fig. 5, *B* and *C*).

Because HOIL-1L is a critical component for the ubiquitination of NEMO, we next tested if the impaired transfer of N-terminally modified ubiquitins was HOIL-1L-dependent. As readout for the functionality of HOIL-1L with different ubiquitins we used the inhibitory effect that HOIL-1L has on the HOIP^RBR-LDD^-mediated linear ubiquitin chain formation (Fig. 4*A* and Fig. 5*D*, *left panel*). Interestingly, HOIL-1L did not affect the ubiquitin chain-forming reaction when the N terminus of the donor ubiquitins was modified with a TAMRA label (Fig. 5*D*, *right panel*). Thus, the N terminus of the donor ubiquitin is essential for the functioning of HOIL-1L in the ubiquitination of NEMO.

The HOIL-1L-dependent steps for NEMO modification, the attenuation of ubiquitin chain formation (Fig. 5*D*), and ubiquitin transfer onto NEMO (Fig. 5, *B* and *C*) rely on an unmodified ubiquitin N terminus of the donor ubiquitin. In contrast, the direct transfer of a donor ubiquitin onto a target ubiquitin by HOIP in linear ubiquitin chain formation is not affected by changes in the donor ubiquitin N terminus (Fig. 3*A* and Fig. 5, *C* and *D*). Thus, the priming ubiquitination on NEMO and linear ubiquitin chain formation place different requirements on the donor ubiquitin.

## DISCUSSION

LUBAC-mediated linear ubiquitin chain formation on NEMO requires a dual target specificity of the complex. First the ubiquitination activity of LUBAC needs to be directed toward a lysine on NEMO, after which the N terminus of this priming ubiquitin on NEMO becomes the target for linear ubiquitin chain extension. We show that the E3-ligase activity that is embedded within the RBR-LDD region of HOIP is essential but not sufficient for the modification of NEMO. HOIP needs the presence of HOIL-1L to not only activate its catalytic core but also to direct the donor ubiquitin toward NEMO. These results are in line with previous studies in cells that show that the NEMO interaction domains in HOIP are redundant when HOIP is in complex with HOIL-1L and that the affinity of the HOIP·HOIL-1L complex for NEMO is higher than of HOIP alone (4).

We used a minimal LUBAC complex consisting of HOIP and HOIL-1L in this study; however, a complex of HOIP and Sharpin also targets NEMO for ubiquitination and has an increased affinity for NEMO in cells compared with HOIP alone (30, 31). Thus, Sharpin may also perform this dual role of HOIP activation and target selection for linear ubiquitin chain formation, although the lack of an RBR in Sharpin indicates that the mechanism would be different. Interestingly, besides NEMO, RIP1 has been identified as a target for linear ubiquitination by LUBAC (9). Therefore, the fact that multiple LUBAC components can activate and direct the activity of HOIP may provide a regulatory mechanism for the target selection of HOIP-mediated linear ubiquitination activity. This suggests that depending on its partner, HOIP activity may be directed to different LUBAC targets.

The relatively slow priming reaction on NEMO by HOIP·HOIL-1L is followed by the much more processive chain-forming activity. The linear ubiquitin chain elongation does not rely on multiple LUBAC components and is controlled by the interplay between the HOIP LDD domain and the target ubiquitin. The position of the N terminus of the target ubiquitin within the ubiquitin structure is essential for the linear ubiquitin chain formation. In addition, the two negatively charged ubiquitin residues E16 and E18 that are positioned next to the N-terminal amino group on the top of ubiquitin are important for the catalysis of the chain formation reaction.

The negatively charged residues on the top of ubiquitin might perform a function within the target ubiquitin to prepare Met-1 for its modification, or ubiquitin E16/E18 may contribute directly to the catalysis of the ubiquitin chain formation by HOIP. The recently solved crystal structures of the RBR proteins Parkin and HHARI revealed the presence of a catalytic triad in the RING2 domain, consisting of Cys-431, His-433, and Glu-444 (numbering for the Parkin sequence) (23–26). In HOIP the corresponding Cys-885 and His-887 are essential for ubiquitin chain formation, whereas Gln-896, which aligns with Parkin Glu-444, is not involved in the chain-forming reaction (25, 28). Therefore, ubiquitin E16 and/or E18 may substitute for the lack of a glutamate in HOIP and might play a direct role in the catalytic mechanism of HOIP to facilitate its specific ubiquitination reaction on the ubiquitin N terminus. Ubiquitin E16 was already shown to play a direct role in the enzyme catalysis of the linear ubiquitin chain-specific de-ubiquitinase Otulin/FAM105B by positioning residues in its catalytic triad in a catalytically active state (39, 40). Thus, the top of ubiquitin contributes to the catalysis of the formation as well as to the destruction of linear ubiquitin chains. Interestingly, an intact N-terminal region also plays an important role in the donor ubiquitin that is priming NEMO.

In sum, the priming lysine ubiquitination on NEMO and the subsequent linear ubiquitin chain elongation are mediated by the catalytic activity of the RBR-LDD region in HOIP, but different additional contributions are required from HOIL-1L and ubiquitin to direct the ubiquitination events toward the different targets.

## Footnotes

2 The abbreviations used are:

NEMO: NF-κB essential modulator
RBR: RING-between-RING
LUBAC: linear ubiquitin chain assembly complex
LDD: linear ubiquitin chain-determining domain
βME: β-mercaptoethanol
TAMRA: 5-carboxytetramethylrhodamine
Ub: ubiquitin.
